# Unique Bacteria Community Composition and Co-occurrence in the Milk of Different Ruminants

**DOI:** 10.1038/srep40950

**Published:** 2017-01-18

**Authors:** Zhipeng Li, André-Denis G. Wright, Yifeng Yang, Huazhe Si, Guangyu Li

**Affiliations:** 1 Jilin Provincial Key Laboratory for Molecular Biology of Special Economic Animals, Institute of Special Animal and Plant Sciences, Chinese Academy of Agricultural Sciences, Changchun, Jilin, 130112, China; 2 School of Animal and Comparative Biomedical Sciences, University of Arizona, Tucson, Arizona, 85721, USA

## Abstract

Lactation provides the singular source of nourishment to the offspring of mammals. This nutrition source also contains a diverse microbiota affecting the development and health of the newborn. Here, we examined the milk microbiota in water deer (*Hydropotes inermis*, the most primitive member of the family Cervidae), reindeer (*Rangifer tarandus*, the oldest semi-domesticated cervid), and the dairy goat (*Capra aegagrus*, member of the family Bovidae), to determine if common milk microbiota species were present across all three ruminant species. The results showed that water deer had the highest bacterial diversity, followed by reindeer, and then goat. Unifrac distance and correspondence analyses revealed that water deer harbored an increased abundance of *Pseudomonas* spp. and *Acinetobacter* spp., while milk from reindeer and goat was dominated by unclassified bacteria from the family Hyphomicrobiaceae and *Bacillus* spp., respectively. These data indicate significant differences in the composition of milk-based bacterial communities. The presence of *Halomonas* spp. in three distinct co-occurrence networks of bacterial interactions revealed both common and unique features in milk niches. These results suggest that the milk of water deer and reindeer harbor unique bacterial communities compared with the goat, which might reflect host microbial adaptation caused by evolution.

Lactation in mammals is an important evolutionary adaption that has resulted from reproductive strategies and developmental requirements. Traditionally, milk is considered to contain bioactive components, macronutrients, and host defense proteins^1^,^2^. However, recent findings have shown that milk, even in the breast tissue of healthy women, is dominated by a complex microbiota^3^,^4^,^5^,^6^,^7^,^8^. The milk microbiota contains potential probiotics while also playing a key role in the initial steps of neonatal gut colonization, the maturation of the infant immune system, and the reduction in the incidence and severity of infections in infants^5^,^9^. Thus, from an evolutionary perspective, it is likely that the milk microbiota is a reflection of host adaptation.

Ruminantia not only is the most important group of large terrestrial herbivorous mammals, but it also significantly diverse, allowing us to better understand the processes of evolution^10^. In relation to family members of this group, Cervidae and Bovidae play important roles in human food and agro-economical products, including meat and milk. Chinese water deer (*Hydropotesthe inermis*) is considered to be the most primitive member of the family Cervidae^10^,^11^, while reindeer (*Rangifer tarandus*) have the longest history of domestication of the Cervid family^2^. Therefore, Cervidae and Bovidae are potentially important when considering an optimal approach to help compare milk microbiota. Moreover, it is likely that different animal host genetic profiles, lifestyles and habitats, and diets may lead to differences in milk composition. Previous studies have demonstrated that the chemical characteristics of cervid milk are different from that of other domestic dairy species^12^,^13^. Reindeer milk has the highest nutritional content (carbohydrate, fat, proteins, branch chain fatty acids, and immune factors) among non-bovine mammals^2^. This has a knock-on effect on the composition of the milk microbiota^7^,^8^,^14^. In addition, water deer milk and reindeer milk are also used as a medical remedy to cure digestive problems^15^. Thus, we hypothesize that the milk of water deer and reindeer harbors unique microbial communities, which to the best of our knowledge have not yet been profiled. Conversely, from a microbial ecology perspective, recent studies have suggested that potentially opportunistic pathogens are inhibited by a cooperative network of human milk bacterial communities. It is likely that these networks are important in the maintenance of milk ecology and health^16^. These findings led us to hypothesize that distinct interactive relationships are at play in milk produced by different species.

In this study, we used next generation sequencing to characterize the milk microbiota of water deer, reindeer, and goat, and to elucidate and compare the interactive relationships of milk bacteria from different species.

## Results

### Sequencing and bacterial community composition in the milk of water deer, reindeer, and goat

Milk bacterial composition was investigated using Illumina MiSeq PE 300 platform sequencing of the 16 S rRNA gene. Eight water deer, nine reindeer, and eight goats were used to facilitate this analysis. Overall, we obtained 499,524 high-quality sequences with an average of 19,980 sequence reads per sample (range 3,596–42,155). Good’s coverage, which is a reflection of sequencing efforts, showed that more than 99% of the bacterial species in the milk from the water deer, reindeer, and goats were captured. Measurements of alpha diversity pertaining to the bacteria (number of observed OTUs, Chao1, Shannon-Weiner and Simpson indices) were significantly higher in the milk of water deer compared to that of reindeer and goat (Figure S1).

**Figure 1:**
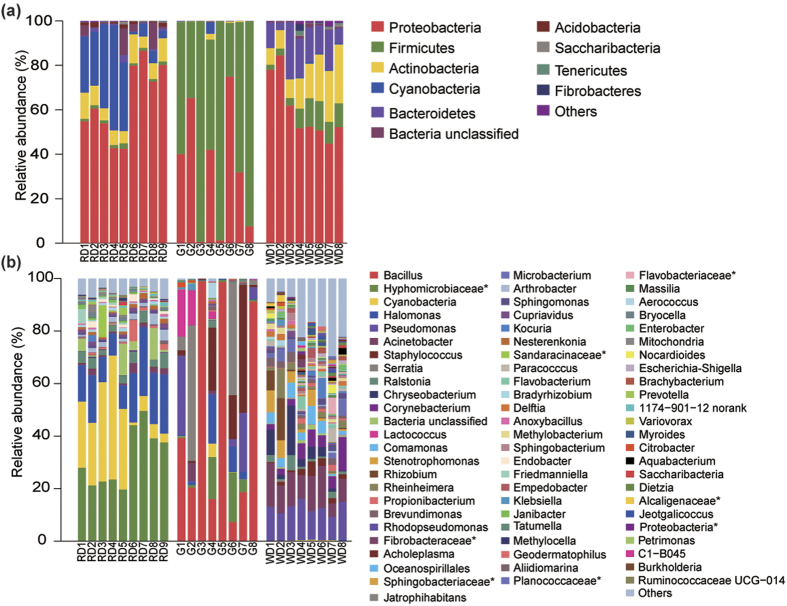
Bacterial composition in the milk of Water deer, Reindeer and Goat at phylum (**a**) and genus (**b**) levels. RD = Reindeer, G = Goat, WD = Water deer. The asterisk means the unclassified bacteria at the family or phylum levels.

Taxonomic assignment of these sequences revealed 26 different phyla of bacteria in the milk (from all three hosts) based on 97% 16 S rRNA gene sequence identity. Bacteria belonging to the Proteobacteria, Actinobacteria, and Bacteroidetes were dominant in the milk of water deer (59.6% ± 14.2%, 15.5% ± 7.3%, and 13.8% ± 6.8%, respectively), whereas bacteria belonging to the Proteobacteria, Cyanobacteria and Actinobacteria were dominant in the milk of reindeer (63.8% ± 16.6%, 20.1% ± 16.8%, and 8.2% ± 2.9%, respectively). Conversely, the milk of goat was dominated by bacteria belonging to the Firmicutes (65.8% ± 29.1%) and Proteobacteria (32.9% ± 28.3%) (Fig. 1a).

At the genus level, the milk of water deer was dominated by *Pseudomonas* spp., accounting for 12.2% ± 2.2% of the observed genera, followed by *Acinetobacter* spp. (11.6% ± 3.9%), *Chryseobacterium* spp. (5.4% ± 3.5%), *Corynebacterium* 1(5.2% ± 3.2%) and *Comamonas* spp. (3.8% ± 2.0%). In the milk of reindeer, unclassified bacteria from the family Hyphomicrobiaceae (31.7% ± 11.1%) were dominant, followed by unclassified Cyanobacteria (20.0% ± 11.8%), and *Halomonas* spp. (16.2% ± 6.3%). The milk of goat was dominated by *Bacillus* spp. (48.9% ± 30.3%), followed by *Serratia* spp. (12.7% ± 7.7%), *Staphylococcus* spp. (11.7% ± 11.1%) and *Pseudomonas* spp. (7.6% ± 3.8%) (Fig. 1b).

### Comparison of milk bacterial composition across all three hosts

**Figure 2:**
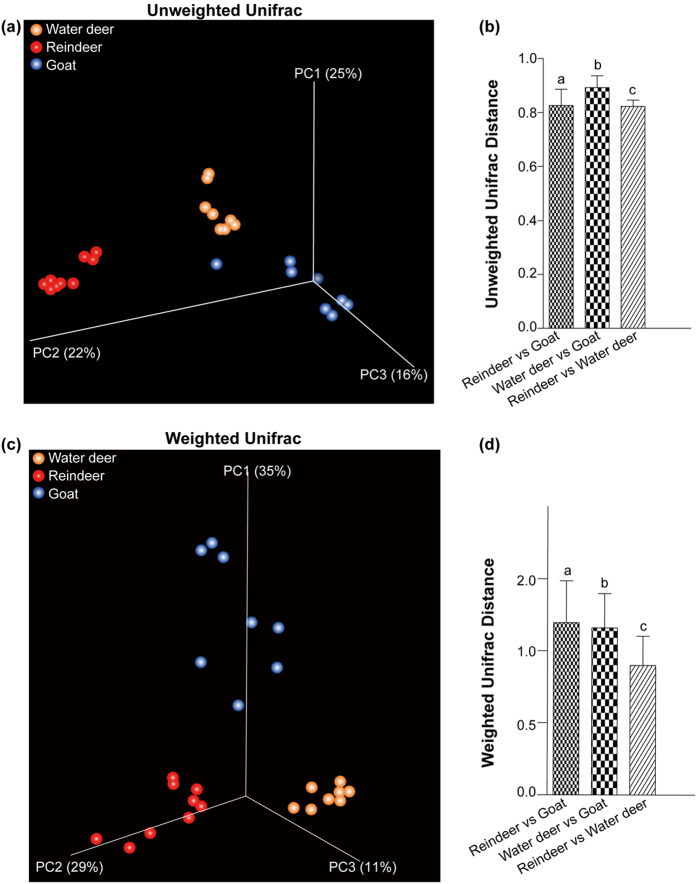
Comparisons of the bacterial communities in the milk of Water deer, Reindeer and Goat. Principal coordinate analyses based on unweighted UniFrac distances (**a**) and average distance differences among groups (**b**). Bar plots labeled with different letters (a, b, and c) denote UniFrac distances between and within three hosts (Water deer, Reindeer, and Goat) are significantly different (Kruskal-Wallis tests, FDR-adjusted q < 0.05). (**c** and **d**) Principal coordinate analyses based on weighted Unifrac distances (**d**) and average distance differences among the three hosts (**d**).

The unweighted UniFrac distance (presence/absence of bacterial taxa) and weighted UniFrac distance (based on presence-absence and relative abundance of bacterial taxa) were applied to examine differences in bacterial community composition and structure across all three hosts. An unweighted UniFrac distance analysis showed that bacterial community composition in the milk of water deer, reindeer, and goat was significantly different (Fig. 2a,b). However, when taking into account bacterial abundance based on a weighted UniFrac distance, the difference in milk bacteria composition between reindeer and goat, and between water deer and goat, were more pronounced (Fig. 2c,d).

**Figure 3:**
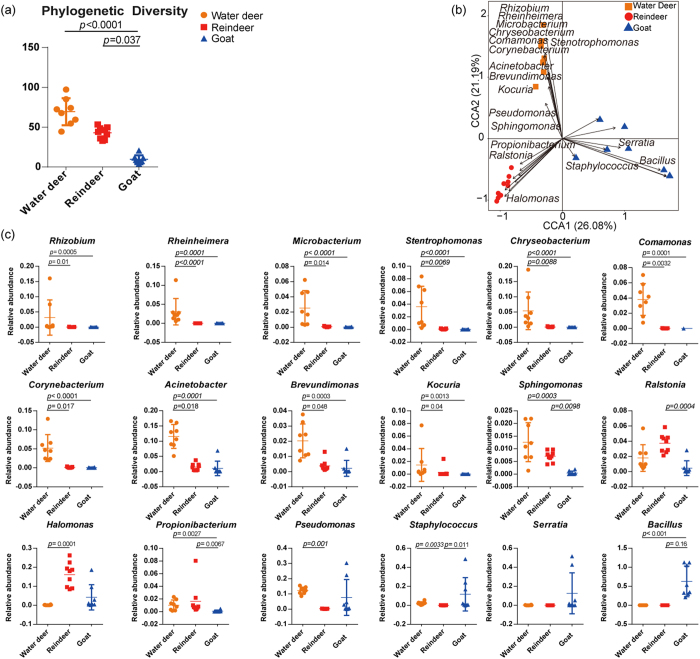
Features characterizing the milk bacteria of Water deer, Reindeer and Goat. (**a**) Phylogenetic diversity comparisons. (**b**) Correspondence analysis (CA) showing indicator genera driving the differences of milk bacteria across all three hosts. The distance between vectors (arrows), and the symbols (circles, squares, and diamonds) that represent each taxon give an estimate of the taxon’s relative abundance in a given sample. (**c**) Boxplots showing differences in the relative abundance of some indicator genera driving milk bacterial differences among the Water deer, Reindeer and Goat.

An exploration of the bacterial features (phylogenetic diversity whole tree) distinguishing all three hosts showed that bacterial diversity in the milk of both water deer and reindeer, was significantly higher than that in goat (Fig. 3a). Moreover, a correspondence analysis based on the abundance of an indicator genus, which was used to characterize each group, was used to identify influential bacteria that facilitated differences across all three hosts (Fig. 3b,c). The milk of water deer was predominantly characterized by *Pseudomonas* spp. (12.2% ± 2.2%), *Acinetobacter* spp. (11.6% ± 3.9%), *Chryseobacterium* spp. (5.4% ± 5.2%), *Corynebacterium* spp. (5.2% ± 3.5%), *Comamonas* spp. (3.8% ± 2.0%), *Rhizobium* spp. (3.1% ± 3.7%), *Rheinheimera* spp. (3.0% ± 3.0%), *Microbacterium* spp. (2.5% ± 2.2%), *Stenotrophomonas* spp. (3.6% ± 3.2%), *Brevundimonas* spp. (2.0% ± 1.1%), *Kocuria* spp. (1.5% ± 2.0%), and *Sphingomonas* spp. (1.3% ± 0.7%). The milk of reindeer was characterized by *Halomonas* spp. (16.2% ± 6.3%), *Ralstonia* spp. (3.7% ± 1.2%) and *Propionibacterium* spp. (1.6% ± 1.4%). Conversely, the goat milk contained a higher abundance of *Bacillus* spp. (48.9% ± 30.3%), *Serratia* spp. (12.7% ± 7.7%) and *Staphylococcus* spp. (11.7% ± 11.1%). Furthermore, we found a gradient distribution in relation to bacterial abundance across all three hosts. For instance, the relative abundance of *Acinetobacter* spp. was increased in the milk of water deer (11.6% ± 3.9%), but was decreased in the milk of reindeer (1.5% ± 1.1%) and goat (1.0% ± 0.8%), respectively. On the other hand, the relative abundance of *Halomonas* spp. was depleted in the milk of reindeer (16.2% ± 6.3%), goat (4.2% ± 4.3%) and water deer (0.1% ± 0.1%), respectively.

### Co-occurrence networks of milk bacteria across all three hosts

**Figure 4:**
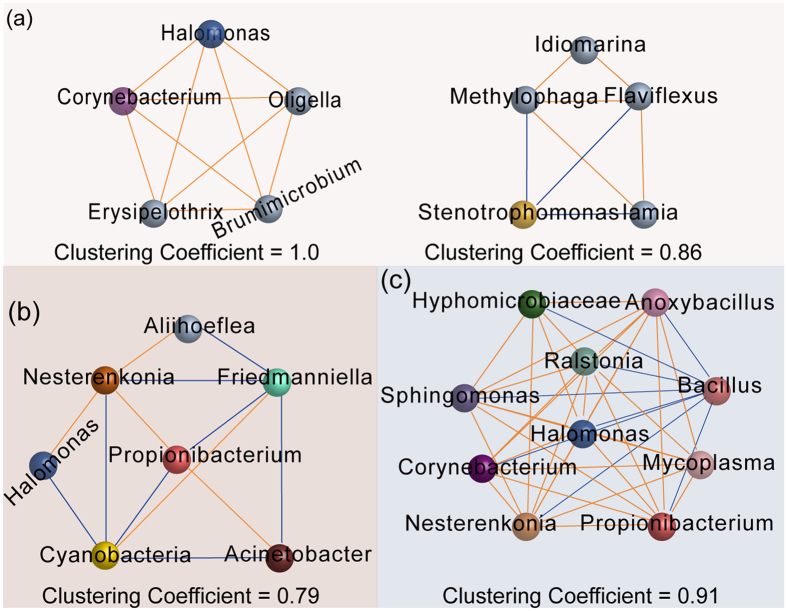
Co-occurrence network of the bacteria in the milk of Water deer (**a**), Reindeer (**b**), and Goat (**c**). Colored circle nodes represent bacterial populations. Each co-occurring pair among bacterial populations has an absolute Spearman rank correlation above 0.90 [Gold line: positive correlation (R > 0.90); Blue line: negative correlation (R < −0.90)] with an FDR-corrected significance level under 0.01.

A network analysis of the milk bacteria at genus level based on Spearman correlation coefficient (R > 0.9) was used to detect co-occurrence patterns (Figure S2). To highlight the biological relevance of bacteria in the milk from all three hosts, we identified clusters (modules) based on a high degree of confidence (Fig. 4). Two modules were identified in the bacterial co-occurrence network in the milk of water deer. *Brumimicrobium* spp. matched the module with the most stable topological structure (clustering coefficient = 1.0), which was positively correlated with *Erysipelothrix* spp. (*p* = 0.001), *Corynebacterium* 1 (*p* = 0.004), *Halomonas* spp. (*p* = 0.00001), and *Oligella* spp. (*p* = 0.004), respectively. Meanwhile, *Idiomarina* spp. matched modules with a clustering coefficient of 0.86 and showed positive correlations with *Methylophaga* spp. (*p* = 0.002) and *Flaviflexus* spp. (*p* = 0.001), respectively. The latter two genera also negatively correlated with *Stenotrophomonas* spp. (*p* = 0.001, and *p* = 0.005, respectively). In the milk of reindeer, we found that *Cyanobacteria* spp. matched modules displaying the most stable coefficient (0.79), and negatively correlated with *Halomonas* spp. (*p* = 0.005), *Nesterenkonia* spp. (*p* = 0.005), *Propionibacterium* spp. (*p* = 0.003), and *Acinetobacter* spp. (*p* = 0.003), respectively. In the milk of goat, unclassified bacteria from *Hyphomicrobiaceae* and *Halomonas* spp. constituted the most matched bacteria, and negatively correlated with *Bacillus* spp. (*p* = 0.002, and *p* = 0.0002, respectively).

## Discussion

In the present study, we observed that bacterial community composition and structure were significantly different across water deer, reindeer, and goat, indicating that host genetics plays a critical role in shaping the composition of milk microbiota. Unique co-occurrence patterns were observed for all three hosts. We also observed the presence of common bacteria, such as *Halomonas* spp., in the networks of all three hosts. Together, these results suggest that the milk of water deer and reindeer harbor unique bacterial community compositions, which is likely to reflect adaptation to different lifestyles.

The results showed that bacterial diversity significantly differed across all three hosts, with the highest diversity observed for the milk of water deer, followed by reindeer and goat (Figure S1). Recent studies have suggested that milk bacterial communities originate from the maternal host or environmental sources including: (i) vaginal or skin bacteria (depending on the delivery model)^17^,^18^; (ii) the infant’s oral and skin bacteria, which may contaminate milk during breastfeeding because of milk flow back into the mammary duct^19^; and (iii) bacteria in the maternal gastrointestinal tract, which translocate through the bacterial entero-mammary pathway into the mammary glands, and then to the milk^20^. However, Hunt *et al*.^3^ have suggested that bacterial communities in the milk of women are not solely the result of skin contamination^3^. Moreover, recent studies have shown that weakened barriers, resulting in increased permeability, and reduced peristalsis, characterize the digestive tract during later pregnancy and lactation^20^. Following the stress of labor and increased gut permeability induced by the delivery process, bacterial translocation from the gut to milk is promoted^17^. Interestingly, water deer (average = 2.3) demonstrated higher reproductive capability compared to reindeer (average = 1)^2^,^11^, and this increased capacity likely causes altered gut permeability. Thus, although our data are based on a limited number of samples, these results indicate that the entero-mammary pathway is an important factor in the occurrence of bacterial diversity in the milk of water deer. These findings prompted us to investigate whether improved reproductive capacity affects the microbiota diversity in milk. We also aimed to elucidate whether the bacterial composition in feces and milk differs between species with different reproductive capacities.

The present study also showed that specific members of bacterial communities were dominant in the milk of the three hosts. *Pseudomonas* spp. and *Acinetobacter* spp. were the predominant bacterial genera in the milk of water deer (Fig. 1b). This finding was not in agreement with previous results pertaining to cow and sheep milk, which both contained a greater abundance of *Lactococcus* spp.^6^,^21^. However, this result was consistent with that of water buffalo, which also showed a relatively high abundance of *Acinetobacter* spp. and *Pseudomonas* spp.^22^. Psychotropic populations, such as *Pseudomonas* spp. and *Acinetobacter* spp., established themselves during cold storage^6^. Sørhaug *et al*.^23^ showed that *Pseudomonas* spp. become the predominant bacterial genus, accounting for up to 70–90% of the bacterial population, when raw milk is stored at low temperatures^23^. Interestingly, water deer (from riverside locations) and water buffalo (deep water) share similar ecological habitat locations^24^. Because these two hosts are genetically distinct, the latter findings suggest that environmental conditions (including temperature and humidity) play an important role in shaping milk bacterial community composition. In addition, factors including health and diet also play a significant role in milk microbiota composition^25^,^26^. This is an area that merits further investigation.

The milk of reindeer contained a disproportionate abundance of unclassified bacteria from the family Hyphomicrobiaceae and *Halomonas* spp. (Fig. 1b). Previous studies have suggested that unclassified bacteria within Alphaproteobacteria are dominant in lichen symbiotic relationships^27^,^28^. Lichens are an important source of energy and nutrients for reindeer in associated habitats. These results indicate that environmental microbial communities (e.g., microorganisms present in diets) are likely to be important contributors to milk bacteria populations in reindeer. *Bacillus* spp. and *Staphylococcus* spp. within phylum Firmicutes, and *Pseudomonas* spp. within phylum gammaproteobacteria were the dominant bacteria in the milk of goat (Fig. 1b). Similarly, McInnis *et al*.^14^ also observed that goat milk contained a high abundance of *Pseudomonas* spp. during early and mid lactation^14^. Moreover, the high proportion of Firmicutes in goat milk correlates with the composition of the gastrointestinal tract of mammals, which is dominated by Firmicutes^29^. Furthermore, the predominant bacterial genera in the milk of goat is similar to those observed in women’s milk, and includes genera such as *Staphylococcus* spp.*, Streptococcus* spp.*, Pseudomonas* spp., and *Lactobacillus* spp. Milk bacteria composition is also similar to that observed for human female breast tissue, which is dominated by *Bacillus* spp.^3^,^7^,^8^,^9^,^17^,^18^,^25^. It is difficult to rationalize the similarity pertaining to the dominant bacteria in the milk of goat and women; however, we speculate that this phenomenon may be related to adaption in relation to civilization in conjunction with the process of domestication.

The bacterial community structures in the milk of both water deer and reindeer were significantly different from that of goat, as revealed by an increased weighted UniFrac distance (Fig. 2). These results reveal that bacterial communities in the milk of water deer and reindeer are more similar to each other than they are to that of goat. Further exploration of the featured bacteria that populate the milk of all three hosts revealed the characterized taxa (Fig. 3). *Propionibacterium* spp. and *Sphingomonas* spp. were prevalent in the milk of water deer and reindeer when compared to that of goat. One plausible explanation is that the proportion of requisite macronutrients, proteins, and immunological factors required by all three hosts is different. Previous studies reported that *Sphingomonas* spp. were the predominant bacteria in the milk of cows exhibiting clinical mastitis^30^. However, these bacteria are also present in the milk of healthy women^3^,^4^. In further, Oikonomou *et al*.^31^ reported that the prevalence of *Sphingobacterium* spp. was associated with increased somatic cell counts in the milk, reflecting the intra-mammary infections^31^. González *et al*.^25^ observed that breast milk containing HIV RNA had a different pattern of microbial composition in comparison with milk lacking HIV RNA^25^. These results suggest that healthy status of mammary glands and immune factors influence milk bacterial composition. Although we provided the different microbiota profile, examining somatic cell counts of milk may provide more insight in the effect of infection and immune function on the milk microbiota.

In addition, the milk of both water deer and reindeer contained *Halomonas* spp. *Halomonas* spp. were identified as the predominant bacteria on the surface of cheese, suggesting that their occurrence may result from proteolysis and salt content, factors which favor the growth of halophilic species^32^,^33^. Metagenomic analysis of milk from women revealed the presence of a significantly higher number of open reading frames related to nitrogen metabolism^7^. Previous results also showed that the milk of cervid animals is enriched with protein, and has a significantly higher casein content than domestic dairy species, including cows, goats and sheep^12^. These results imply that nutritional content is an important determinant in bacterial community structure. Together, these results suggest that the bacterial community structure of milk is determined by milk composition and quality characteristics, such as nutritional content and immunological factors. These factors require further investigation to determine if a definitive relationship exists between microbial composition and milk-specific characteristics.

The co-occurrence network generated as part of this analysis exhibited distinct bacterial community membership across all three hosts (Fig. 4), indicating that the milk microbial ecosystem may depend on different interactive relationships. *Pseudomonas* spp. were the predominant bacteria in the milk of water deer, and did not play a role in maintaining the interactive relationships in the network. The reason why *Pseudomonas* spp. are not functional in the network may be due to the relatively high diversity pertaining to the milk of water deer. A previous study demonstrated that the presence of dominant species could result in a negative relationship between species diversity and ecosystem function^34^.

The co-occurrence analyses also showed that *Halomonas* spp. presented in the networks of all three hosts, and *Propionibacterium* spp. presented in the networks of reindeer and goat. *Halomonas* spp. were observed to interact with *Corynebacterium* spp. and *Nesterenkonia* spp. in the networks. *Corynebacterium* spp. are characterized by proteolytic activity and the production of volatile sulfur compounds or ammonia^35^. In addition, genome analysis of *Nesterenkonia* spp. showed that these bacteria are adapted to extreme environments, exhibiting ammonia assimilation and nitrate/nitrite ammonification capacities^36^. Although the beneficial characteristics of *Halomonas* spp. are still unclear, their frequent detection in cheese^32^,^37^ indicates a specific role in fermentation. However, a recent study showed that both *Corynebacterium* spp. and *Halomonas* spp. displayed positive growth responses on cheese curd agar under deacidification^38^. These results indicate that the interactive roles exhibited by *Halomonas* spp. are determined by the composition of the milk environment.

On the other hand, Ma *et al*.^16^ also revealed the presence of cooperative *Propionibacterium* spp. in women’s milk. Interestingly, *Propionibacterium* spp. isolated from dairy environments have been reported to play health-promoting roles, fermenting lactate into propionate, acetate, and carbon dioxide, thereby resulting in the lipolysis of branched chain acids following the catabolism of amino acids^39^. Moreover, a genomic analysis of *Propionibacterium freudenreichii* demonstrated the presence of a number of genes encoding surface proteins, which were potentially involved in adhesion and immune-regulatory activities, and β-galactosidase-mediated breakdown of lactose^6^,^40^. These results suggest that beneficial bacteria may play a key role in the milk ecology of ruminant animals through interaction with other bacteria. Conversely, we observed that *Bacillus* spp. negatively interacted with other bacteria in the milk of goat. *Bacillus* spp. are important milk spoilage organisms, causing off-flavoring and curdling, and the production of different types of toxins^41^. These findings are consistent with ideas postulated by Ma *et al*.^16^, who initially suggested that opportunistic pathogens including *Staphylococcus* spp. and *Corynebacterium* spp. could be inhibited through bacterial interactions, which are ultimately important in determining milk characteristics^16^. These results indicate the important role of bacterial interactions in relation to ecology and function.

## Conclusion

In the present study, we examined the composition of bacterial communities in the milk of water deer, reindeer, and goat. The results demonstrated that bacterial diversity was significantly different across all three hosts. Moreover, the different milks were dominated by distinct bacterial community structures and compositions. A co-occurrence analysis of bacteria revealed that there were different interactive patterns among the three hosts, but common features were also observed, including the presence of *Halomonas* spp. Moreover, opportunistic pathogens including *Bacillus* spp. were inhibited by other bacteria. Overall, these results indicate that the milk of water deer and reindeer harbor unique and different bacterial communities, which may reflect the occurrence of milk-specific microbial adaptation on an evolutionary timescale.

## Materials and Methods

### Animals and milk samples

Water deer (*Hydropotes inermis*, n = 8) were maintained at a local farm in Yancheng city, Jiangsu province, China (33.20°N, 120.50°E), which were fed sweet potato leaf and tofukasu. The semi-domesticated reindeer (*Rangifer tarandus*, n = 9) in this study were distributed in the Greater Khingan Mountains in the Inner Mongolia autonomous region, China (50.77°N, 121.47°E), grazing the later fall pasture, mainly comprised of mosses and lichen. These animals are maintained by local farmers. The goats (*Capra aegagrus*, n = 8) in the study were maintained at a research farm in the Institute of Special Animal and Plant Sciences, Chinese Academy of Agricultural Sciences, in Jilin province, China (44.04°N, 129.09°E). Goats were fed the same diet consisting of alfalfa and concentrate, containing corn and soybean meal, once in the morning and once in the evening. All animal-specific procedures were approved and authorized by the forestry bureau of Jiangsu province and Inner Mongolia autonomous region, the Chinese Academy of Agricultural Sciences Animal Care and Use Committee, and the Institute of Special Animal and Plant Sciences Wild Animal and Plant Subcommittee. All methods were carried out in accordance with the approved guidelines and regulations.

Before milk sample collection, teats were dipped in iodine, followed by alcohol wipes. Raw milk was collected manually from each animal during the early lactation (about 20 ml) period each morning. The milk samples were immediately placed in liquid nitrogen, and were stored at −80 °C for later analysis.

### DNA extraction and next generation sequencing

Total genomic DNA was extracted from microorganisms in the milk using a PowerFood microbial DNA isolation kit (MoBio Laboratories Inc., Carlsbad, CA) according to the manufacturer’s instructions. A total of 1 ml of raw milk was used for each extraction procedure. The V1–V3 region of the bacterial 16 S rRNA gene was amplified using primers 27F^42^ and 519R^43^. Each specific primer pair contained the appropriate Illumina adapter sequence, and an 8-bp barcode. The resultant amplicons were purified using a QIAquick PCR Purification Kit (QIAGEN, Valencia, CA). The purified amplicons were then sequenced on an Illumina PE MiSeq 300 platform generating paired 300 bp paired-end reads.

### Sequence and bioinformatics analyses

The read pairs were extracted and concatenated according to the barcodes for each paired read resulting in the generation of contigs. Contigs with an average quality <20 over a 10 bp sliding window were culled. After removing low-quality sequences, the retained sequences were processed and analyzed using QIIME 1.7.0^44^. In brief, the sequences were clustered into operational taxonomic units (OTUs) using UPARSE and 97% sequence identity^45^. Potential chimera sequences were removed using UCHIME^46^. The representative sequences of the OTUs were assigned against the SILVA database (version 123) using the RDP classifier with a 0.80 confidence threshold^47^,^48^. The phylogenetic tree was constructed using FastTree^49^. Because the diversity metrics are sensitive to sampling effort, we rarefied the data to the lowest sequencing effort (3,500 sequences). Alpha-diversity of each sample including Chao1, Shannon and Simpson indices, Good’s coverage, phylogenetic diversity and UniFrac distances were subsequently calculated post-rarefaction using QIIME 1.7.0^44^.

### Statistical analysis

To compare the bacterial communities in the milk across all three hosts, the unweighted UniFrac distances (which facilitate an investigation into the presence and absence of bacterial lineages) and weighted UniFrac distance (which takes relative abundances of bacterial lineages into account) were used to perform the principal coordinate analysis (PCoA). All multivariate and community analyses were performed using the reshape, ggplot2, coin, exactRankTests and stats packages implemented in the R (http://www.R-project.org/). Kruskal–Wallis analysis was used to test the statistical significance of alpha-diversity indices and the relative abundance of each taxon in all three hosts. Significance (*p* < 0.05) was based on the Benjamini-Hochberg corrected *p*-value from the Kruskal–Wallis test. All values were expressed as the mean and standard deviations (mean ± S.D) unless otherwise stated.

Indicator species analysis in the milk bacteria of each host was performed using the RAM package^50^. The indicator species analysis selected the most representative features for each cluster or group and split these features into the number of clusters being compared. Associated taxa were chosen by assigning an indicator value to each taxon. This indicator value was the product of the relative average abundance and relative frequency of that feature in a group. Kruskal-Wallis tests adjusted for multiple comparisons were used to confirm the significance of these indicator species. The co-occurrence network analysis was used to examine the existence of true correlations among the milk bacteria across all three hosts according to our previous method^51^. In general, spearman’s rank correlations and *p*-values were calculated and plotted using the hmisc and corrplot packages. Statistical *p*-values were corrected using the Benjamini-Hochberg method. Correlations have an absolute spearman’s correlation greater than 0.9 with a corrected significance level less than 0.01. Network analyses were carried out with Cytoscape 2.8.2 using a force-directed algorithm^52^. Highly connected bacterial clusters (modules) in the network were identified using Network Module identification (NeMo)^53^. The mining of high-confidence modules was largely dependent on the attainment of high scores using NeMo and high clustering coefficients within modules (clustering coefficients closer to 1.0 represent higher fidelity, with the highest being 1.0).

## Additional Information

**Accession codes:** The sequences in the present study were deposited in the SRA database under accession number SRP075175.

**How to cite this article**: Li, Z. *et al*. Unique Bacteria Community Composition and Co-occurrence in the Milk of Different Ruminants. *Sci. Rep.* **7**, 40950; doi: 10.1038/srep40950 (2017).

**Publisher's note:** Springer Nature remains neutral with regard to jurisdictional claims in published maps and institutional affiliations.

## Supplementary Material

Supplementary Information
