# Supplementary material for: Unique Bacteria Community Composition and Co-occurrence in the Milk of Different Ruminants
Source: Sci Rep. 2017 Jan 18;7:40950. doi: 10.1038/srep40950 (PMC5241872; doi:10.1038/srep40950)
Supplement: Supplementary Information [file srep40950-s1.pdf]

Supplementary Information

Unique Bacteria Community Composition and Co-occurrence in the Milk of Different Ruminants

Zhipeng Li[a,#], André-Denis G Wright[b], Yifeng Yang[a], Huazhe Si[a], Guangyu Li[a,#]

a. Jilin Provincial Key Laboratory for Molecular Biology of Special Economic Animals, Institute of Special Animal and Plant Sciences, Chinese Academy of Agricultural Sciences, Changchun, Jilin, 130112, China

b. School of Animal and Comparative Biomedical Sciences, University of Arizona, Tucson, Arizona, 85721, USA

Corresponding authors

Guangyu Li, Email: tcslgy@126.com; Tel: 8643181919805, Fax: 8643181919800

Address: No. 4899, Juye Street, Jingyue District, Changchun, Jilin, 130112, China.

Zhipeng Li, Email: zhplicaas@163.com; Tel: 8643181919515, Fax: 8643181919800

Address: No. 4899, Juye Street, Jingyue District, Changchun, Jilin, 130112, China.

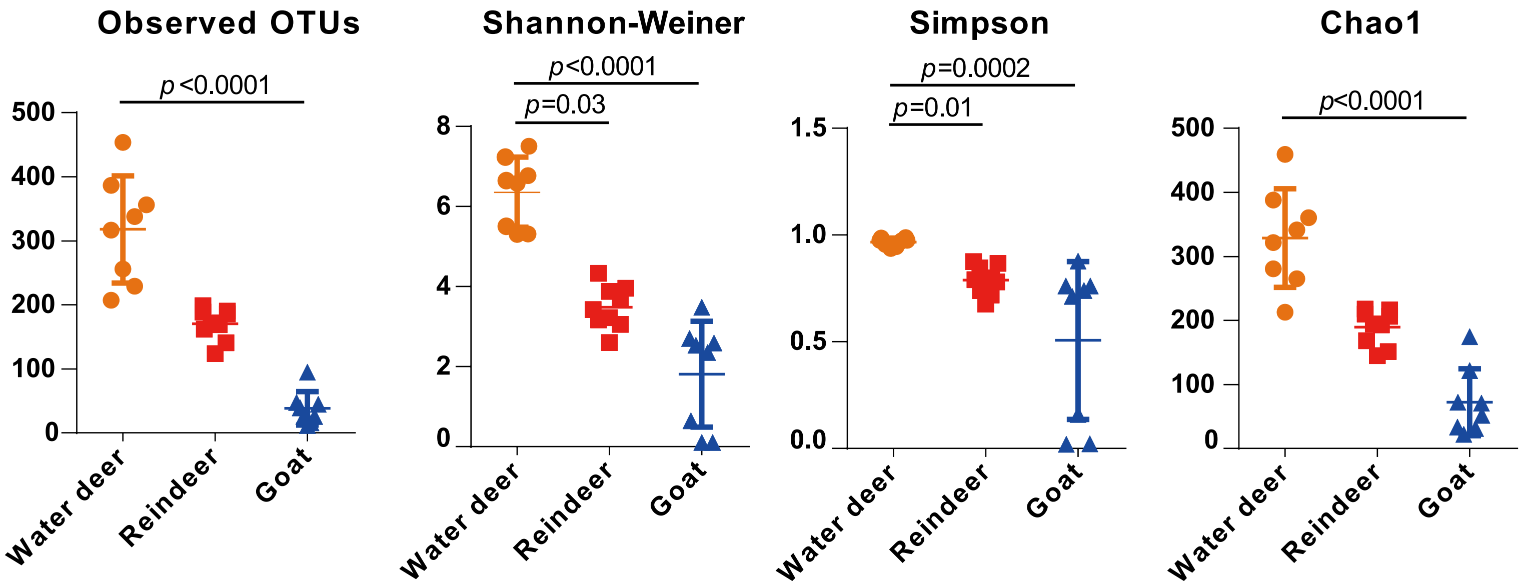

Figure S1. Comparison of the diversity and richness indices across all three hosts.

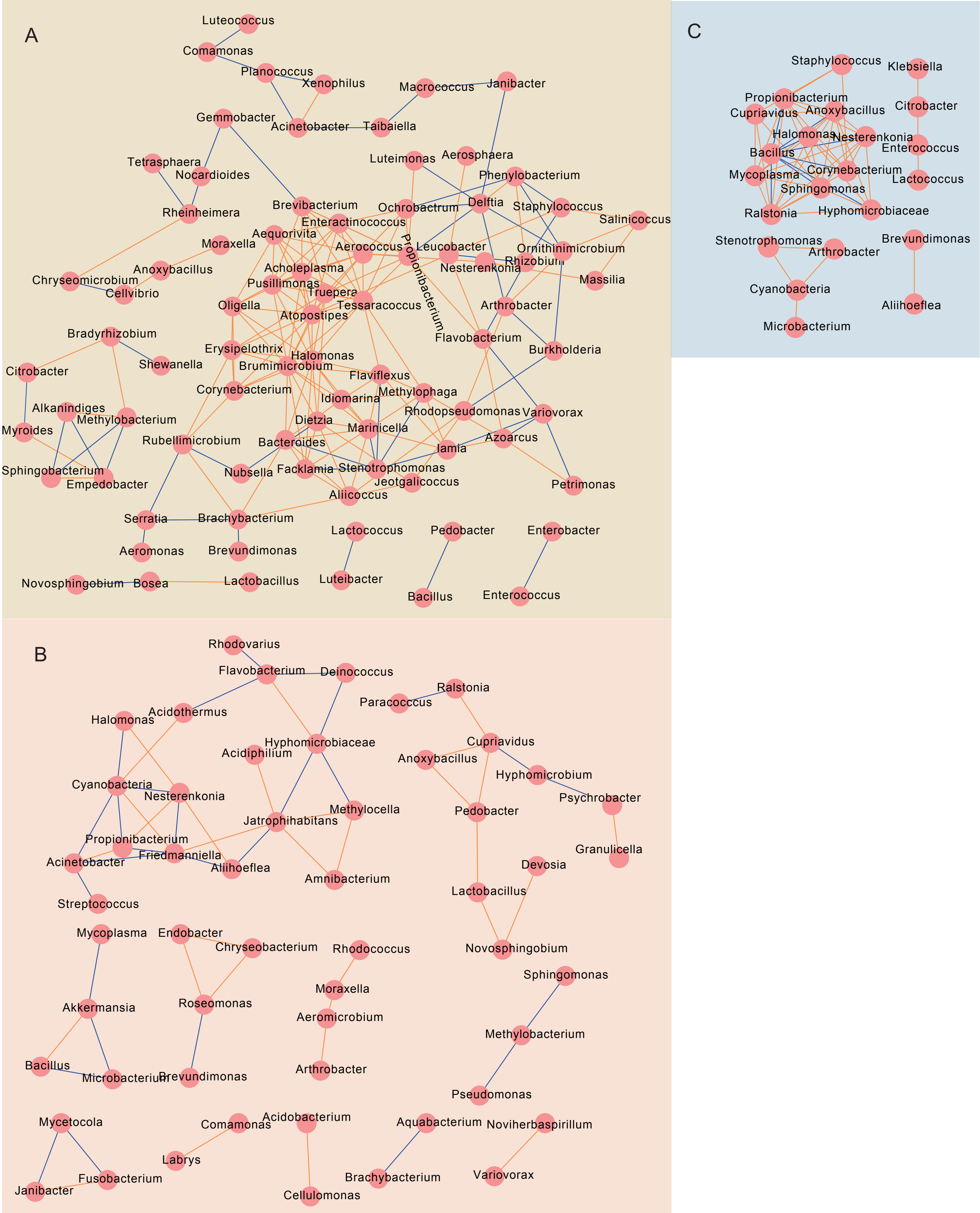

Figure S2. Co-occurrence network of the bacteria from the milk of Water deer (A), Reindeer (B) and Goat (C). Circle nodes represent bacterial populations. Each co-occurring pair among bacterial populations has an absolute Spearman rank correlation above 0.90 [Gold line: positive correlation R > 0.90); Blue line: negative correlation (R < -0.90)] with an FDR-corrected significance level less than 0.01.
